# Comparison of sustained-release morphine with sustained-release oxycodone in advanced cancer patients

**DOI:** 10.1038/sj.bjc.6601365

**Published:** 2003-11-25

**Authors:** G R Lauretti, G M Oliveira, N L Pereira

**Affiliations:** 1 Faculty of Medicine of Ribeirão Preto, Rua-Campos Sales, 330, Apto. 44, University of São Paulo, São Paulo 14015-110, Brazil; 2 Pharmaceutical Technology of the Faculty of Pharmaceutical Sciences of Ribeirão Preto, University of São Paulo, Brazil

**Keywords:** controlled-release oxycodone, controlled-release morphine, cancer pain

## Abstract

The antinociceptive effect of morphine and oxycodone is mediated preferentially at *μ* and *κ* receptors, respectively. The aim of this study was to evaluate the analgesic profile of the combination of morphine and oxycodone in cancer pain, compared to the standard administration of morphine alone. Controlled-release formulations of oxycodone (CRO) and morphine (CRM) were compared in 26 patients. The study started with an open-label, randomised titration phase to achieve stable pain control for 7 days, followed by a double-blind, randomised crossover phase in two periods, 14 days each. At any point, patients were allowed to use oral immediate-release morphine (IRM) as needed, in order to keep visual analogue scale ⩽4. Pain, satisfaction, adverse effects and number of daily rescue morphine tablets were assessed. A total of 22 patients were evaluated. The weekly upload consumption ratio in morphine/oxycodone was 1 : 1.8 (1.80, 1.83, 1.76, 1.84). The weekly IRM consumption was higher in patients having CRM compared to patients having CRO (ratio morphine/oxycodone: 1.6, 1.6, 1.6, 1.7) (*P*<0.05). Patients receiving oxycodone complained of less nausea and vomiting. The rescue morphine analgesic consumption was 38% higher in patients receiving only morphine, compared to patients receiving both morphine and oxycodone. The results suggest that the combination of morphine/oxycodone (opioids with differential preferential sites of action) can be a useful alternative to morphine alone, resulting in a better analgesia profile and less emesis.

Controlled-release morphine (CRM) or controlled-release oxycodone (CRO) are among the pharmacological alternatives in the management of chronic cancer pain. Although both drugs have been used regularly, each drug has been individually evaluated, and as a final result, each patient has normally used either morphine or oxycodone alone (Heiskanen and Kalso, 1997; Mucci-LoRusso *et al*, 1998; Heiskanen *et al*, 2000; Klepstad *et al*, 2003). Among the strategies for the treatment of cancer pain, there is interest in the coadministration of opioids that act on different receptors (Heiskanen *et al*, 2000; Ripamonti and Dickerson, 2001). For instance, the antinociceptive effect of morphine and the semisynthetic opioid oxycodone appears to be mediated preferentially at *μ* and *κ* receptors, respectively (Ross and Smith, 1997; Nielsen *et al*, 2000), and oxycodone may offer enhanced analgesia when combined with morphine.

The aim of this study was to evaluate the analgesic profile of the combination of the opioid morphine and oxycodone in chronic cancer pain, compared to the standard administration of morphine alone. Immediate-release morphine (IRM) was used as an escape analgesic in order to obtain the information related to the interaction morphine/oxycodone.

## MATERIALS AND METHODS

The Ethical Committee of the University of São Paulo's Teaching Hospital, Ribeirão Preto, approved the study protocol. Controlled-release formulations of oxycodone and CRM were evaluated in 26 patients with chronic cancer pain of the visceral and somatic type (either oropharynx, lung, colon, gastric, ovary or prostate gland, as described in Table 1

**Table 1:** Origin of cancer in the population studied

**Origin of cancer**	**Number of patients**
Oropharynx	9
Lung	3
Colon	4
Gastric	2
Ovary	2
Prostate gland	2
	
Total no. of patients	22

), after informed consent of the patients. The concept of visual analogue scale (VAS), which consisted of a 10 cm line with 0 equalling ‘no pain at all’ or ‘no nausea’, and 10 equalling ‘the worst possible pain’ or ‘worst possible nausea’ was introduced previously. Before enrolling in this actual study, patients received 3–4 mg kg^−1^ tramadol, plus nonsteroidal anti-inflammatory drugs; however, they still complained of pain VAS ⩾4 cm. As part of the protocol, all patients were taking oral 25 mg amitriptyline at bedtime.

The study was started with an open-label, randomised titration phase to achieve stable pain control for 7 days. At this initial phase, patients used only IRM and had free access to it in order to keep pain VAS <4 cm. After stable pain relief was achieved, this was followed by a double-blind, crossover phase in two periods, 14 days each. Each patient acts as his/her own control to minimise the interindividual variability of response in this group of patients, and no period of washout was allowed for ethical reasons. At this phase, patients did not know which treatment they were enrolled in. The optimum opioid dosage was calculated on a daily basis, and the consumption ratio of oxycodone to morphine was set at 1 : 1.8, as part of the study protocol. In the literature, this ratio has varied from 1 : 1; 1 : 2; 2 : 3; 3 : 4 (Heiskanen and Kalso, 1997; Zhukovsky *et al*, 1999; Hanks *et al*, 2001). Immediate-release morphine was used as an escape analgesic during the second phase (double blind) of the study in order to get the information related to the morphine/oxycodone interaction.

Patients were randomised to receive either CRO (14 days) followed by CRM (14 days) (*n*=13), or the same drugs in inverse order (*n*=13), and were followed on a weekly basis. The doses of either CRM or CRO were assigned daily by the pharmaceutical, who was aware of the drugs and treatment, and set at 1 : 1.8 by the same pharmaceutical (one of the authors). The tablets of IRM were substituted by the respective controlled-release formulation, always by this same investigator. The anaesthesiologists who collected the data weekly were blind to the treatments during the crossover phase. At any point, patients were allowed to use oral IRM (10 mg tablets) as an escape analgesic, as needed, in order to keep the numeric value of the pain VAS ⩽4 cm. Patients were asked to assess pain intensity, patient satisfaction, adverse effects and number of rescue morphine tablets, and a different investigator unaware of the treatments, recorded the data weekly. All patients were cooperative and understood the protocol.

The final collected data were evaluated for each period (14 days) for each patient, related to the consumption of IRM, and to the final ratio of the weekly CRO/CRM, in order to check the initial assumption of the relation of morphine/oxycodone to be 1 : 1.8, made always by the pharmaceutical not involved in data collection. At the end of the study, every patient had received (1) CRM and IRM (the morphine-alone phase), and (2) CRO and IRM (the combined phase). The phase entitled ‘morphine alone’ received only morphine as analgesic during the 14-day period. The phase entitled ‘combined’ received both oxycodone and morphine during the 14-day period. The IRM consumption was indicative of the analgesic interaction between morphine and oxycodone.

### Statistical analysis

The statistical analysis for the opioid consumption was performed using the Mann–Whitney *U*-test and the Wilcoxon signed-rank test. Adverse effects were compared using the *χ*^2^ test. Significance was set at *P*<0.05.

## RESULTS

Of the 26 patients enrolled in the study, 22 were evaluated. Withdrawals were due to death unrelated to the study (one patient), uncontrollable nausea and vomiting (one patient), and unstable pain control requiring spinal drugs (two patients). The population was 59±19 years old, 52±8 kg of weight, 163±7 cm of height and the male : female ratio was 15 : 7. The number of patients receiving concomitant radiation therapy or chemotherapy during the study, and the origin of cancer are described in Tables 1 and 2

**Table 2:** Number of patients having adjuvant therapies

**Adjuvant therapies**	**Number of patients**
Radiation	1
Chemotherapy	6
Radiation/chemotherapy	4
None	11
	
Total no. of patients	22

. Although eight patients complained of emesis while taking only morphine (CRM and IRM), three of them refereed dissatisfaction and would prefer oxycodone because of the high frequency of vomiting, which was not related to chemotherapy. The total of five patients enrolled in radiation therapy had it regularly throught the study protocol.

**Figure 1:**
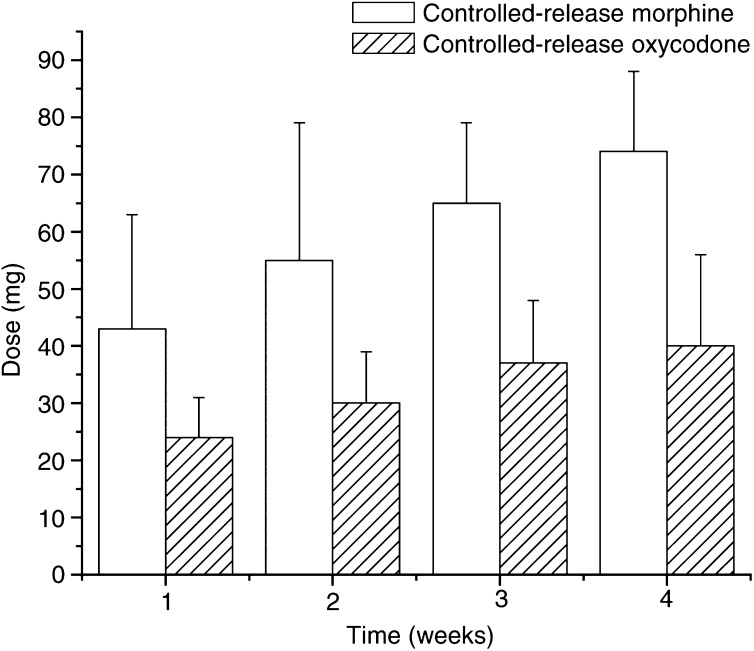
Mean final weekly doses of CRM and CRO (mg) are described in bars. The lines over the bars indicate the standard deviation of the mean. The consumption ratio of CRM/CRO was 1.80, 1.83, 1.76 and 1.84, during the study period, set by the pharmaceutical.

The weekly opioid consumption ratio of morphine/oxycodone was set on a weekly basis at 1 : 1.8 in all phases (mean 1.80, 1.83, 1.76, 1.84) by the pharmaceutical. The mean final weekly dose of morphine and oxycodone (mg) is described in Figure 1

. When the results of the two phases were compared, the patients consumed 38% more IRM when using CRM and IRM, compared to the ‘combined phase’ (i.e. when patients received both CRO and IRM). The range of morphine daily consumption (mg) for the morphine-alone phase was: first week 20–60 mg; second week 30–90 mg; third week 40–90 mg; and fourth week 60–90 mg. The range of oxycodone daily consumption (mg) for the combined phase was: first week 20–40 mg; second week 20–40 mg; third week 20–60 mg; and fourth week 20–60 mg.

**Figure 2:**
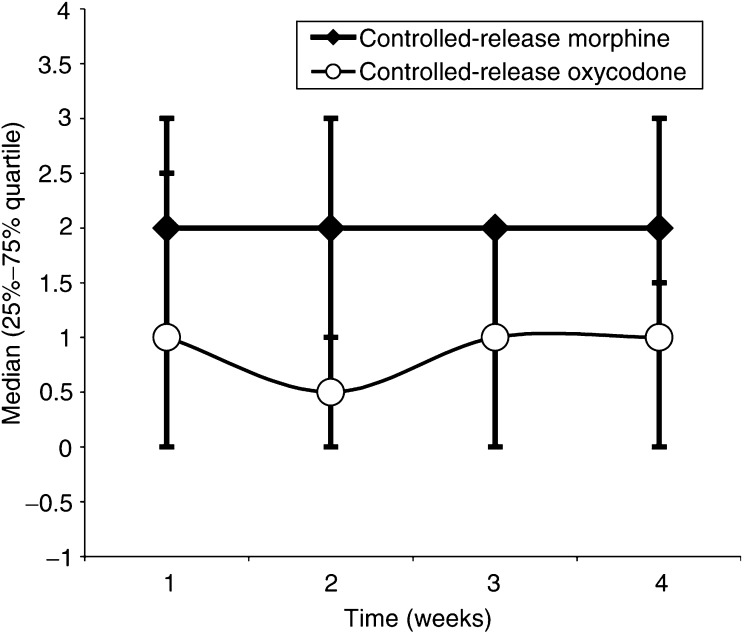
Mean daily number of rescue analgesic IRM (10 mg) at the end of each week. The weekly IRM consumption was higher in patients having CRM compared to patients having CRO (*P*<0.05). Morphine values: 2(0–2.5); 2(0–3); 2(0–2); 2(1–3). Oxycodone values: 1(0–3); 0.5(0–2); 1(0–2); 1(0–1.5).

The mean daily IRM consumption was higher in patients having CRM compared to patients having CRO, independent of which opioid drug was administered first (morphine/oxycodone ratio 1.6, 1.6, 1.6, 1.7) (Figure 2

) (*P*<0.05). The daily pain VAS was less than 4 cm in all patients, as part of the study design, and not different among the phases (*P*>0.05).

Related to side effects, patients receiving oxycodone complained of less nausea and vomiting, compared to patients receiving morphine only (*P*<0.05). The incidence of dry mouth, somnolence, hallucination, constipation, pruritus, sensation of empty head, anorexia, dyspnoea and good acceptance to the study drugs was not different among patients, independent of the phases (Table 3

**Table 3:** Number of patients complaining of adverse effects and acceptance to the study drugs

	**Combined phase**	**Morphine-alone phase**
Nausea*	1	8
Vomiting*	0	7
Dry mouth	3	2
Hallucination	0	0
Somnolence	7	11
Pruritus	1	1
Constipation	4	5
Sensation of empty head	1	0
Anorexia	14	13
Dyspnoea	0	0
Acceptance to the study drugs	22	21
Patient satisfaction	22	18

; *P*>0.05). There were no reports of hallucinations or dyspnoea in either group.

## DISCUSSION

The results of the study have demonstrated that patients suffering from cancer pain receiving the combination of morphine and oxycodone consumed significantly less escape doses of IRM, which was 38% higher in patients receiving morphine only. The data suggest that the combination of morphine/oxycodone (Ross and Smith, 1997; Nielsen *et al*, 2000) can be a useful alternative to morphine alone, resulting in a better analgesia profile, which is in accordance with animal studies. Coadministration of subantinociceptive doses of oxycodone with morphine to rats by both intracerebroventricular and systemic routes (intraperitoneal and subcutaneous) resulted in synergistic levels of antinociception (Ross *et al*, 2000). Behaviourally, rats coadministered subantinociceptive doses of oxycodone and morphine were not different from control rats related to sedation, while showing antinociception (Ross *et al*, 2000).

In accordance with the World Health Organisation guidelines for cancer pain relief, when initiating treatment, controlled-release preparations of opioids are generally favoured, and are combined with IRM to prevent or treat breakthrough pain (Enting *et al*, 2001). In the present study, the optimum opioid dosage was calculated on a daily basis, and the consumption ratio of oxycodone to morphine was set at 1 : 1.8, as part of the study protocol. In the literature, this ratio has varied from 1 : 1, 1 : 2, 2 : 3, 3 : 4 (Heiskanen and Kalso, 1997; Zhukovsky *et al*, 1999; Hanks *et al*, 2001). Washout periods cannot be used for ethical reasons, and a better design including immediate-release oxycodone could not have been carried out due to its unavailability in Brazil. However, the absence of a washout period was overcome by the fact that half of the patients had started the medication with oxycodone, and the other half had started with morphine. Adjuvant therapies that could affect pain control, such as radiation and chemotherapy, were carried out throught the protocol by the patients involved, as detailed in Table 1, and should not interfere with the final results, as every patient worked as his/her own control.

As part of the protocol, all patients were exposed to IRM prior to randomisation to CRM or CRO, potentially producing tolerance. Opioids that interact with *μ*- and/or *κ*-binding sites demonstrate an adaptation process described as desensitisation, due to a reduced interaction with the internal second-messenger system of G-protein (Freye and Latasch, 2003). Repeated stimulation of *κ*-opioid receptors leads to the heterologous upregulation of *μ*-opioid receptor functions in the thalamus and periaqueductal grey regions, which may be associated with the supersensitivity of *μ*-opioid receptor-mediated antinociception (Narita *et al*, 2003), and *κ*-receptors may be involved in multiple mechanisms in the mesencephalon (Sun and Dalman, 2003). As a consequence, the possibility of tolerance development during the first open phase would rather interfere with patients taking oxycodone, an opioid with a preferential site of action at *κ* receptors. In spite of this, the final data revealed that it probably did not occur at the time the study was conducted, based on the lesser IMR consumption in the combined phase.

Despite the preferential action at *κ* receptors (Ross and Smith, 1997; Nielsen *et al*, 2000), oxycodone is an opioid analgesic that closely resembles morphine. Oxymorphine, the active metabolite of oxycodone, is formed in a reaction catalysed by the cytochrome isoenyme CYP2D6, which is under polymorphic, genetic control and severely impaired by liver dysfunction. However, the role of oxymorphone in the analgesic effect of oxycodone is not yet clear (Heiskanen *et al*, 2000). Although gender differences exist in response to oxycodone either due to pharmacodynamics or differences in metabolism related to reduced CYP2D6 in females (Davis *et al*, 2003), in the present study, each patient participated of both study groups, and acts as his/her own control, minimising any analgesic tendency in the female population. In addition, an unidentified metabolite other than oxymorphone appears to be a potent *μ* agonist (Poyhia *et al*, 1993), and the intrinsic efficacy of oxycodone that may not correlate with binding affinity is not known (Duttaroy and Yoburn, 1995).

Unlike oxycodone, the active metabolite of morphine, morphine-6-glucorinide, appears to have a better toxicity profile and a similar analgesic effect compared to morphine (Cann *et al*, 2002). While patients received CRM, the consumption of IRM was 38% higher compared to the combination group, suggesting a better profile of the association of opioids with preferentially binding sites at *μ* and *κ* receptors (Nielsen *et al*, 2000), such as morphine and oxycodone.

Nevertheless, pain relief and side effects such as emesis, sedation, itching and hallucinations have been described following morphine administration, while being less frequent at equianalgesic doses after oxycodone (Heiskanen and Kalso, 1997; Mucci-LoRusso *et al*, 1998). In the actual study, when patients received the combination of oxycodone and morphine, they complained of less nausea and vomiting, in accordance with others (Heiskanen and Kalso, 1997), while constipation has been reported to be more frequent after oxycodone alone (Heiskanen and Kalso, 1997). Side effects such as dry mouth, somnolence, constipation, pruritus, sensation of empty head, and good acceptance to the study drugs were not different among patients in the present study (Table 3). Nevertheless, group sizes in the range of 30–60 would be more appropriate for accuracy of side effects (Moore *et al*, 1998).

In conclusion, the rescue morphine analgesic consumption was 38% higher in patients receiving morphine only, compared to patients receiving both morphine and oxycodone, suggesting that the combination of morphine/oxycodone (opioids with differential preferential sites of action) can be a useful alternative to morphine alone, resulting in a better analgesia profile and less emesis. Unfortunately, the cost of CRO treatment in Brazil would be nearly three times more expensive than morphine alone.
